# Assessment of Energy Expenditure of Polish Special Forces as a Determinant for Planning the Energy and Nutritional Value of Daily Food Rations

**DOI:** 10.3390/nu18132167

**Published:** 2026-07-03

**Authors:** Paweł Kler, Anna Anyżewska, Karolina Bertrandt, Roman Łakomy, Andrzej Tomczak, Sebastian Sobczuk, Karolina Jamka, Jerzy Bertrandt

**Affiliations:** 1 Faculty of Security, Logistics and Management, Military University of Technology, gen. Sylwestra Kaliskiego 2, 00-908 Warsaw, Poland; pawel.kler@wat.edu.pl; 2 School of Medical & Health Sciences, University of Economics and Human Sciences in Warsaw, Okopowa 59, 01-043 Warsaw, Poland; 3 Military Institute of Aviation Medicine, Krasinskiego 54/56, 01-755 Warsaw, Poland; 4 Independent Researcher, 05-506 Lesznowola, Poland; 5 WSB Merito University in Poznan, 61-895 Poznan, Poland; 6 Doctoral School, Military University of Technology, gen. Sylwestra Kaliskiego 2, 00-908 Warsaw, Poland; 7 Faculty of Economic Sciences, John Paul II University in Biala Podlaska, Sidorska 95/97, 21-500 Biala Podlaska, Poland; jwbertrandt@gmail.com

**Keywords:** nutritional needs, energy expenditure, nutrition security, food ration

## Abstract

**Background/Objectives:** Human nutritional requirements are closely linked to energy expenditure, which depends on daily and occupational activities. Studies on groups performing tasks requiring increased physical effort may support determining their energy and nutritional needs. The study population consisted of soldiers performing a wide range of tasks domestically and during missions abroad, whose physical effort may be comparable to that of athletes in demanding sports disciplines. The aims of this study were (1) to assess the energy expenditure of soldiers performing training tasks in two different special forces units, as a basis for evaluating the physical demands of their work; (2) to evaluate the daily energy expenditure value as a basis for planning the energy and nutritional value of the daily food ration, as well as to develop a proposal for a nutritional standard dedicated to collective feeding in special forces units. **Methods:** The study included soldiers from the Special Unit “GROM” and from the Special Branch of the Military Police (MP). Energy expenditure was measured using heart rate monitoring and analysis of heart rate variability. **Results:** The average daily energy expenditure related to field training was 4175 ± 723.7 kcal for GROM soldiers and 5014.8 ± 666.3 kcal for soldiers of MP. **Conclusions:** To ensure safe and adequate nutrition for special forces soldiers, the energy value of the daily food ration—after applying a 5–10% safety margin—should be no less than 4400 kcal. Considering the significant increase in energy expenditure during intense training, the average value of this increase was determined to be approximately 500 kcal. It was proposed to increase the energy value of the daily food ration by 500 kcal. Based on the findings, nutritional requirements were determined as a proposal for a basic nutritional standard for soldiers of Polish special units.

## 1. Introduction

Energy expenditure associated with various training and service-related tasks performed by soldiers is a key indicator of total daily energy needs. Understanding these values enables appropriate regulation of physical workloads during training and helps determine optimal daily caloric requirements.

The training process for special forces soldiers differs significantly from that of soldiers in other branches of the military. Although the physical activity involved is similar to the training of athletes in high-intensity sports [1,2,3,4], it is characterized by a distinct specificity. While the physical activity of athletes is a planned, systematic, and targeted training process aimed at maximizing performance in a chosen discipline [5,6,7], Special Forces training involves multi-hour sessions conducted at unusual times of the day and in unfamiliar environments. Above all, the type of tasks to be performed is unknown, and there is a constant potential for encountering unforeseen difficulties that result in an increased energy load. During training and exercises, soldiers carry out tasks including unconventional operations, specialized reconnaissance, direct-action missions, and anti-terrorism activities [8]. Soldiers of special units of the Polish Armed Forces not only carry out training activities during garrison and field exercises but also perform a wide variety of missions during deployments abroad.

Although some selective studies have examined the impact of individual gear weight and marching pace on the energy expenditure of Polish special forces soldiers in recent years [9], an assessment of the daily energy expenditure of soldiers in the special units of the Polish Armed Forces has never been conducted. Consequently, the value of energy expenditure resulting from the training process and the nature of service in this type of military units remains unknown. While data exist regarding the energy expenditure of US special forces soldiers [10,11], their results cannot be directly applied to the soldiers of the Polish Armed Forces special units due to a different training process and different equipment. Therefore, it became necessary to evaluate the energy expenditure of the Polish Armed Forces special units, the results of which can provide a basis for assessing the physical demands associated with training and service, and subsequently allow for the regulating of this workload according to the physical capabilities of the soldiers. Based on the obtained results, it will be possible to determine the daily energy expenditure associated with training and service in this type of units, which will provide a basis for determining the energy value of the daily food ration that balances this expenditure and ensures safe and adequate nutrition. Since the Polish Armed Forces currently lack nutritional standards for special forces soldiers, the results obtained from this study will allow for the development of the energy and nutritional value of a daily food ration dedicated to soldiers of this branch of the armed forces.

Several special units operate within the Polish Armed Forces, including the GROM Military Unit and the Special Unit of the Military Police (MP).

The GROM Unit is an elite force trained for operations across diverse environments. It is capable of executing a broad range of special missions, such as conducting special operations in land, sea, and air domains, counter-terrorism operations within Poland and abroad, hostage rescue, protection of high-value individuals and infrastructure, evacuation of personnel from high-risk areas, including diplomatic facilities, etc.

The Special Unit of the Military Police is responsible for protecting the senior officials of the Ministry of Defense, ensuring the security of key state officials while they are present on military premises, and safeguarding members of foreign delegations visiting Poland.

Given the nature of the tasks and the specific demands of service, proper nutrition becomes especially critical for special forces personnel. In other words, soldiers must be provided with nutrition security. This is defined as ensuring that food is harmless to humans, properly stored, prepared for consumption, and consumed in appropriate portions, while meeting all the body’s nutritional needs. Therefore, their food rations must cover the high energy demands associated with the training process and provide all essential nutrients in the correct amounts and proportions. Ultimately, guaranteeing nutrition security is one of the most important factors in maintaining soldiers’ health and peak physical performance, and it must be continuously adapted to changing operational conditions and needs [12].

The training program for special forces includes not only general military instruction but also specialized training tailored to specific mission profiles. Special Forces soldiers in Poland receive rations according to the basic feeding standard 020 [13] and the supplementary feeding standard 110 [13], regardless of their training intensity. The energy value of this nutrition is estimated at 3800–4100 kcal. To date, the Polish Armed Forces have not conducted studies to assess the actual energy expenditure of Special Forces soldiers. Therefore, the energy expenditure associated with their training and service has not been previously established. Historical data from other armed forces suggest that the energy demands of special forces personnel are very high, ranging from approximately 4099 kcal (17.1 MJ/day) in the US Army Ranger School [14] to 5198 kcal (21.7 MJ/d) during Special Forces selection for the US military [15].

Given the broad range of tasks performed by Polish special units as part of national and international military operations, it has become necessary to conduct research to assess energy expenditure during training and service. The outcomes of this research will form the basis for designing food rations tailored to actual energy and nutritional requirements, ensuring safe and effective nutrition. In Poland, collective feeding is based on two types of standards: nutritional standards, which reflect the actual physiological requirements for energy and nutrients, and feeding standards, i.e., standards for the supply of energy and nutrients calculated on the basis of the energy value and the content of individual nutrients in food products included in the technological recipes of daily food rations. It should be emphasized that there are currently no nutritional standards in the Polish Armed Forces that would cover soldiers’ physiological requirements for energy and all nutrients resulting from training processes, the specific nature of military service, and the type of unit in which the service is performed. At present, feeding standards are used in the planning and provision of meals for soldiers of the Polish Armed Forces. In these standards, the energy and nutritional value of daily food rations is determined using a calculation-based method based on the energy value and the content of individual nutrients in food products included in the technological recipes of food rations, rather than on the actual energy expenditure associated with the nature and specific characteristics of military service. Therefore, it is assumed that determining the energy expenditure of soldiers of special units of the Polish Armed Forces will provide the basis for developing a proposed nutritional standard dedicated to this group of soldiers. Thus, in practice, soldiers of the Polish Armed Forces are provided with meals according to feeding standards in which the energy and nutrient content of rations is based on the caloric values of individual food items used in meal planning—rather than on the actual energy expenditure associated with the nature of service. At present, Polish Special Forces’ peacetime nutrition is based on the basic feeding standard 020, which provides 4532 kcal (18.9 MJ) [13,16].

The study pursued two objectives: (1) to assess the energy expenditure of soldiers performing training tasks in two different special forces units, as a basis for evaluating the physical demands of their work; and (2) to evaluate the daily energy expenditure value as a basis for planning the energy and nutritional value of the daily food ration, as well as to develop a proposal for a nutritional standard dedicated to collective feeding in special forces units.

## 2. Materials and Methods

### 2.1. Participants

A total of 114 male soldiers participated in the study, including 36 from the Special Unit “GROM” and 78 undergoing training in the Special Unit of the Military Police. The characteristics of the soldiers participating in the study are presented in Table 1. The research was conducted in the course of typical activities associated with military training and service duties. Due to the specific nature and execution of the training tasks, in many cases, it was not possible for the research team to directly monitor the training process.

This study was conducted in accordance with the Declaration of Helsinki of the World Medical Association, and approved by the Ethics Committee of the Military Institute of Hygiene and Epidemiology (5 December 2016, Protocol No 1/XXI/2016). Participants were provided with an information sheet outlining the study’s purpose, procedures, and potential risks and benefits. All participants gave their informed consent.

### 2.2. Measurement of Height and Weight

Body height (without shoes) was measured using a portable stadiometer, TANITA HR-001 (Tanita Corporation, Tokyo, Japan) [17]. Body weight was assessed using the bioelectrical impedance analysis (BIA) method with the TANITA MC-780 device (Tanita Corporation, Tokyo, Japan) [18], with a precision of 0.1 kg, in accordance with the user manual (participants were lightly clothed and barefoot). All measurements were conducted according to the manufacturer’s instructions and without any metal objects on the body.

### 2.3. Measurement of Energy Expenditure

Energy expenditure was measured using heart rate monitoring and analysis of heart rate variability. This approach is widely used and effective under conditions of intense field operations; however, it is subject to certain limitations arising from individual variations in the relationship between heart rate and oxygen consumption, the influence of environmental factors, and potential measurement interference. Although special forces soldiers operate not only under physical exertion but also in conditions of prolonged, multifactorial stress, including sleep deprivation, limited energy availability, load carriage, exposure to adverse environmental conditions, and the need to make decisions under pressure, this is the only method currently approved by senior command for assessing energy expenditure during official tasks included in the training program. The method relies on the linear relationship between heart rate (HR) and oxygen uptake (VO_2_) during physical activity. The assessment involved recording heart rate using the Polar RC3 GPS heart rate monitor (Polar Electro Oy, Kempele, Finland) [19]. Polar monitors are used to estimate daily energy expenditure across varying intensities of activity and are well-suited for use in field conditions [20]. The device calculates energy expenditure based on the relationship between heart rate and the energy cost of work, in accordance with ISO 8996:1990E [21]. The reported value is the average of multiple readings. Previous studies have shown that these devices offer relatively high accuracy, with results in healthy individuals closely matching those obtained simultaneously with electrocardiograms (correlation coefficient *r* = 0.99) [22]. Additionally, device validation was performed using the MWE-1 energy expenditure meter (CB Electronics, Warsaw, Poland), which evaluates energy output by measuring the subject’s forced expiratory airflow and correlating ventilation volume with oxygen uptake to produce an energy expenditure value [23]. The conducted validation studies demonstrated that the energy expenditure measurements obtained from the device did not differ significantly from those calculated based on oxygen consumption using standard laboratory equipment under light, moderate, heavy, and very heavy exercise conditions, with a correlation coefficient (*r* = 0.99) [23].

Before testing, individual heart rate and VO_2_ calibration curves were established for each participant. Individual calibration curves were established based on heart rate measurements and oxygen consumption during incremental exercise performed on a cycle ergometer [24]. Energy expenditure during soldiers’ task performance was measured three times, and the final result was taken as the average of these three measurements. Energy expenditure values for common daily activities—such as sleeping, washing, cleaning, exercising, eating, watching television, and others—were derived from standard energy expenditure tables for Polish soldiers serving in various military branches [25]. The study was conducted during both garrison-based and field training. The resulting data were used to determine the energy demands associated with the nature and specificity of military service and to classify the workload intensity according to Lehman’s [26] or Christensen’s [27] classification systems (Table 2 and Table 3).

### 2.4. Statistical Analyses

All statistical analyses were performed using R software, version 4.4.0 [28]. Anthropometric data are presented as mean values ± standard deviation. Differences between the experimental groups were analyzed using ANOVA and Tukey’s post hoc tests, with statistical significance set at *p* ≤ 0.05. The Shapiro–Wilk test was applied to assess the normality of variable distribution, while Levene’s test was used to verify the homogeneity of variances. Differences in energy expenditure values between GROM soldiers and those from the Special Unit of the Military Police were determined using the Chi-square (χ^2^) test.

## 3. Results

### 3.1. Assessment of Nutritional Status

Analysis of anthropometric results revealed no significant differences between soldiers from the respective units, with the exception of height—MP soldiers were found to be taller (Table 1).

### 3.2. Assessment of Energy Expenditure During Training

Energy expenditure was assessed during typical training activities carried out under garrison conditions as outlined in the training schedule. These included measurements of energy expenditure during lectures, firearms training with various types of weapons, and physical education sessions. In contrast, energy expenditure associated with field training was assessed during standard tactical exercises and special operations. The results of energy expenditure measurements are summarized in Table 4 and Table 5.

Based on the results obtained, the average energy expenditure of GROM unit soldiers undergoing training under garrison conditions over an 8-h training day was 1521.6 kcal, classifying their activity as heavy work. In contrast, the energy expenditure of soldiers from the Special Unit of the Military Police was 2064 kcal, which qualifies as very heavy work.

The study showed that energy expenditure during 8 h of tactical and shooting training in field conditions amounted to 1872 kcal for soldiers of the Special Unit GROM, classifying the workload as heavy. In contrast, soldiers of the Special Unit of the Military Police expended 2304 kcal, placing the intensity of their work in the very heavy category.

**Figure 1:**
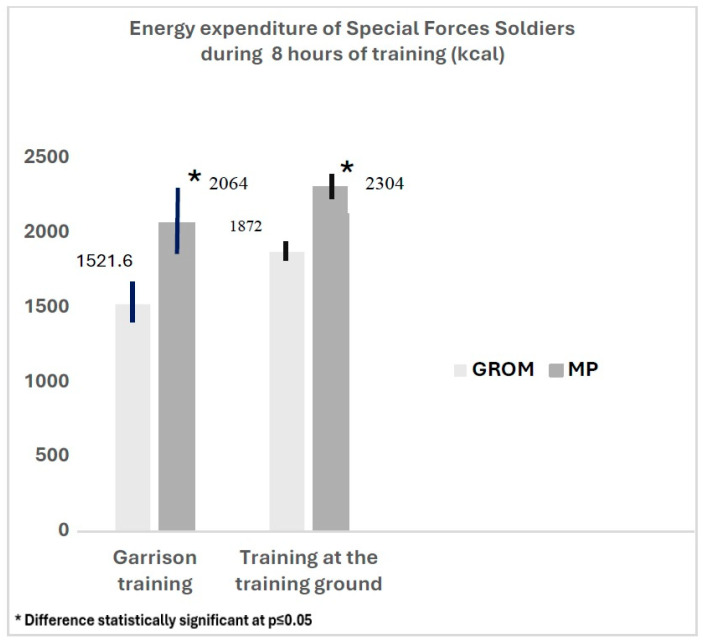
Energy expenditure values of soldiers of the Special Unit GROM and the Special Unit of the Military Police.

The findings indicate that energy expenditure associated with the execution of training tasks were significantly higher in the Special Unit of the Military Police (MP) compared to the Special Unit GROM, during both garrison-based and field training (Figure 1).

GROM unit soldiers also undergo training in maritime environments. Energy expenditure during training was assessed in two groups of soldiers participating in specialist exercises—either onboard boats or involving underwater operations. The results showed that energy expenditure associated with 8-h specialist training under maritime conditions was high and amounted to:

- 2482 ± 235 kcal/8 h for soldiers trained on boats;
- 2376 ± 134 kcal/8 h for soldiers carrying out underwater tasks.

Considering a standard 8-h shift, the average energy expenditure for soldiers training under maritime conditions was 2429 ± 184.5 kcal, categorizing this type of work as very heavy.

Energy expenditure was also measured during 24-h training exercises conducted in field conditions. The study involved 22 male soldiers from the Special Unit GROM, with a mean age of 30.1 ± 3.1 years, average body weight of 86.8 ± 10.0 kg, and height of 179.8 ± 6.6 cm. Daily energy expenditure was measured three times for each soldier, during three separate 24-h field training sessions. These intensive exercises lasted between 18 and 20 h and included activities such as overcoming terrain obstacles, performing cross-country runs, and shooting with various types of weapons. Sleep duration during the 24-h period ranged from 2 to 4 h. Daily energy expenditure values are presented in Table 6.

It was found that 24-h tactical training generated an average energy expenditure of 4175.0 ± 723.7 kcal/day per soldier. According to Lehman’s classification, this places the intensity of their work in the heavy workload category [26]. While the average daily energy expenditure indicates that soldiers were performing heavy work, it is important to note that the energy expenditure was determined by the nature, type, and environmental conditions of each task. In many cases, daily energy expenditure exceeded 4500 kcal, and in some instances reached extreme values of up to 5700 kcal/day.

The daily energy expenditure study related to field training involved 26 male soldiers serving in the Special Unit of the Military Police. Their average age was 32.5 ± 5.9 years, with a mean body weight of 86.9 ± 8.8 kg and a mean height of 181.5 ± 5.8 cm.

Table 7 presents the energy expenditure values of MP soldiers during typical daily activities performed under field training conditions.

### 3.3. Assessment of the Daily Energy Balance of Soldiers Trained in Field Conditions

The basic principle of rational nutrition is to maintain a balanced systemic energy balance. The daily energy balance refers to the comparison between the amount of energy supplied to the body through food and the amount of energy expended by the body over the course of a day. It is a key factor affecting body mass and overall health. Soldiers of the Special Forces of the Polish Armed Forces, when trained in field conditions, are covered by a collective feeding system in accordance with feeding standard 020, which provides for a daily energy supply of 4535 kcal per food ration [16]. Considering the daily energy expenditures associated with training tasks in the field—4175.0 kcal for soldiers of the Special Unit GROM and 5014.8 kcal for soldiers of the Special Unit of Military Police—a positive daily energy balance of +360 kcal was recorded for the former group, while the latter exhibited a negative daily balance of −479.8 kcal. It is important to note that both positive and negative energy balances have adverse effects on soldiers’ nutritional status. Excessive energy intake can lead to weight gain and an increased risk of diet-related lifestyle diseases. Conversely, prolonged energy deficits can result in weight loss and a decline in physical performance. Nutritional imbalances negatively affect the performance of both training and operational duties and may lead to the disqualification of soldiers from service—particularly in special forces units.

## 4. Discussion

Conducting special operations in demanding terrain and climatic conditions requires individuals with exceptional personal and physical qualities, verified during specialized exercises, and capable of executing difficult, risky, and dangerous missions. Special forces soldiers represent a military group that routinely engages in prolonged, strenuous field operations, resulting in a consistently high level of daily physical activity [10,29] and an energy expenditure that generally exceeds that of the average soldier [11,30]. The groups of soldiers analyzed in this study, through multi-stage training, must acquire specific skills appropriate to athletes practicing the most physically demanding disciplines, such as combat sports [2,31]. Exceptional traits acquired during long-term training include: excellent physical fitness and the ability to energy management, resistance to fatigue in extreme environmental conditions, the ability to cope with stress and pain, and the use of combat strategy [32,33]. Achieving systemic energy balance during field operations is challenging, as calorie intake is often restricted to short, dispersed intervals, and food availability is limited to what the soldier can carry [34]. During extended periods of intense physical activity and constrained food supplies, it becomes increasingly difficult to achieve sufficient calorie intake to offset expenditure, leading to a negative energy balance [35].

In peacetime, the primary mission of special forces soldiers is intensive training. The training process differs significantly from that of soldiers serving in other branches of the armed forces. Commandos assigned to special operations must possess specific physical and mental endurance, and be accustomed to working under varying intensities and timeframes.

The analysis of training programs for special forces soldiers usually does not allow, as it does in standard military units, for a precise hourly breakdown of training activities. This is because their training process involves diverse tasks without assigning specific time durations to each. It is worth noting that a training day for special forces personnel rarely follows a structured “daily schedule” with clearly defined time slots for activities, meal breaks, or rest periods. Given that many soldiers in these units return home after completing a typical day of training, it was only possible to measure the energy expenditure of individual activities performed during training and then convert these into an estimate of average hourly energy use (i.e., over the course of a working shift). As a result, the assessment of total daily energy expenditure was carried out only during field training exercises, where training activities lasted 18 h or more.

In a study conducted by W. Tharion on U.S. special forces soldiers undergoing garrison-based training, the average total daily energy expenditure was reported as 4099 ± 740 kcal/d [30]. Another study, conducted by L. M. Margolis et al., aimed to determine the energy expenditure of U.S. special forces soldiers during a typical military training program [10]. During the observation period, 31 participants engaged in daily physical training, including group runs of up to five miles, rigorous calisthenics, four hours of intensive pool training, open-water swimming, and other exercises. The total daily energy expenditure amounted to 4204.31 ± 555.45 kcal.

The average energy expenditure of U.S. special forces soldiers—4099 ± 740 kcal/d—falls within the military reference energy standards, between high activity (3700 kcal/d) and exceptionally high activity (4700 kcal/d) [36].

According to the recommendations on calorie and nutritional values and the composition of wartime rations intended for NATO Response Forces [37], two types of food rations are used: one for scheduled training conditions and one for wartime conditions. Their respective calorie values are 3600 kcal/d and 4900 kcal/d [38].

The energy expenditure values obtained for GROM unit soldiers—4175.0 ± 723.7 kcal/d—are consistent with results from studies on U.S. special forces and with NATO recommendations [38].

Training in field conditions is always associated with greater energy expenditure compared to garrison-based training. This is due not only to differences in the training program itself but also to significant environmental influences such as temperature, terrain, and weather, as well as the extension of training hours to 16—and in special units—even up to 20 h per day. Daily energy expenditure values reported in various sources generally cluster around 4500 kcal/d [39].

Earlier studies on the energy load of soldiers among different branches of the Polish Armed Forces showed that garrison training in the Land Forces generated an average energy expenditure of 2251 kcal per 8-h day, with a daily expenditure of 4375 kcal/d. In field conditions, the daily expenditure was slightly higher at 4583 kcal/d. For comparison, the daily energy expenditure of Polish Navy crews during a typical training day was 3908 kcal, while that of Air Force flying personnel on flight days was 3274 kcal/d [40].

The training activities of Special Forces soldiers are generally associated with high energy expenditure due to sustained physical exertion. Most studies concerning energy expenditures in special units focus on the preparatory phase—that is, periods of intensive training typically conducted under field conditions. In line with the findings presented in this study, other research on the calorie needs of soldiers serving in Special Forces units indicates that their needs are higher than those necessary to maintain the energy balance of soldiers serving and training in other branches of the military. Variations in reported energy expenditures can be attributed to differences in environmental conditions, the volume and intensity of exercise, characteristics of the studied population, sample size, duration of the study, and the methods used for parameter assessment, and also depends on the gender of the participants [11,30].

The results of energy expenditure studies conducted among soldiers from two Polish special forces units are consistent with findings obtained for U.S. special forces soldiers and those of other nations.

Previous studies have shown that energy expenditure among U.S. Army Special Forces personnel ranged from approximately 4100 kcal/d (17.1 MJ/d) during Ranger School training [14] to around 5200 kcal/d (21.7 MJ/d) during recruitment and selection courses for U.S. Special Forces [15]. The level of physical activity observed during training of U.S. Special Forces exceeded the upper threshold of sustainable energy expenditure (>2.5) [29], which, without proper nutritional intervention, can lead to body mass loss and potentially impair physical performance [41].

Data published in 2014 on energy expenditure during basic training courses for U.S. special forces soldiers—conducted both on land and at sea—were similar to the values obtained in the present study. The soldiers’ energy expenditure was 3901 ± 520 kcal/d (16.3 ± 2.1 MJ/d) and 4564 ± 350 kcal/d (19.1 ± 1.4 MJ/d), respectively. It was found that a standard ration designed for stable operational conditions provided approximately 3250 kcal, which was insufficient to meet the calorie demands of the training process [10].

Kyröläinen et al. demonstrated that during a 7-day military exercise involving daily marches of 20–25 km through forest terrain, with full equipment and weaponry weighing a total of 49.8 ± 4.7 kg, soldiers expended 7000 kcal/d (29.3 MJ/d) [42]. Other studies have shown that the energy expenditure of U.S. special forces soldiers trained in field conditions reached 5185 kcal/d. It was further demonstrated that total energy expenditure increased by 15% when compared to garrison training—rising from 4350 kcal (18.2 MJ) to 5210 kcal (21.8 MJ) [43]. Additional research on daily energy expenditure during training of U.S. special forces soldiers revealed a daily expenditure of 4099 kcal, which exceeded the caloric value of the food rations provided—3950 kcal [16]. As a result, it is recommended that calorie intake from rations used for feeding U.S. Special Forces soldiers be increased to 125% of the base ration’s calorie value [44].

Numerous studies have indicated that military training in mountainous terrain may result in energy expenditures ranging from 14.8 to 29.8 MJ/d [11]. Extreme values related to the implementation of special operations in the mountains were recorded among soldiers of French mountain special units during a 29-h, highly intensive high-altitude training exercise. These soldiers were found to expend 43.57 ± 1.23 MJ/d [45].

Most previous studies on physical activity and energy expenditure among special forces soldiers have focused on candidates undergoing initial selection courses [46,47]. These physically demanding courses often include food restriction, sleep deprivation, and exposure to extreme environmental conditions—deliberately designed to push participants to their physical limits, ensuring that only the strongest and most resilient complete the selection process. It has been suggested that nutritional recommendations for special forces personnel, based on findings from such selection courses, may be overestimated, and that the actual calorie demand of soldiers engaged in operational and training duties may be lower. Only a limited number of studies examining energy expenditure among special forces soldiers have been conducted under garrison conditions [30]. Available literature indicates that energy expenditure associated with military training across various armed forces ranges from 3109 kcal/d (13 MJ/d) to 7131 kcal/d (29.8 MJ/d) [11], with field training typically resulting in values well above 3000 kcal/d (12.6 MJ/d) [48]. A study of energy expenditure among Zimbabwean soldiers performing field tasks in a hot climate revealed a daily energy output of 5497 kcal [49]. This elevated expenditure was strongly influenced by tropical environmental conditions. Other studies have shown that energy expenditures of special forces personnel conducting training in hot and cold environments were similar—4664 ± 1399 kcal/d and 4549 ± 1221 kcal/d, respectively. The average energy expenditure was 4618 ± 1350 kcal/d, while the caloric content of the daily food ration was 2429 ± 838 kcal, resulting in a negative energy balance of approximately 2200 kcal/d [50].

Taking into account that daily energy expenditure constitutes the basis for planning the energy and nutritional value of the daily food ration, the study led to the determination of the energy requirements for soldiers of special forces units and, consequently, to the recommended intake of protein (13–15% of energy), fats (25–30% of energy), and carbohydrates (55–62% of energy), as well as vitamins and minerals in accordance with current nutritional standards in Poland. The study also resulted in a proposal for a basic nutritional standard for soldiers of special forces units of the Polish Armed Forces.

## 5. Proposal of the Basic Nutritional Standard for Special Units of the Polish Armed Forces

Proposed basic nutritional standard for soldiers of special units of the Armed Forces of the Republic of Poland is presented in Table 8.

## 6. Limitations

This study has certain limitations. The most important of these is the training process of soldiers of special units, who perform tasks of various nature under diverse climatic and environmental conditions and are required to make immediate decisions depending on the situation. This generates a high level of stress and may significantly affect the level of energy load. Another limitation is the small number of participants performing specific training tasks, since soldiers from this type of unit usually operate in small teams. An important limitation is also the research method, since in studies involving this group of soldiers, the only feasible method is heart rate monitoring, which does not interfere with the training process and does not pose a risk to the health of participants during the performance of non-standard training tasks.

## 7. Conclusions

The results of the study indicate that the average energy expenditure associated with an 8-h training day under garrison conditions was 1521.6 kcal for soldiers of the Special Unit GROM and 2064 kcal for soldiers of the Special Unit of the Military Police. Under field training conditions, these values increased to 1872 kcal and 2304 kcal per 8-h period, respectively. The average daily energy expenditure during field training reached 4175.0 kcal among GROM soldiers and 5014.8 kcal among soldiers of the Special Military Police Unit. These findings demonstrate that the training activities performed by soldiers of both units generally correspond to heavy or very heavy physical work. Consequently, to ensure safe and adequate nutrition for special forces personnel, the energy value of the daily food ration, including a 5–10% safety margin, should not be lower than 4400 kcal. Furthermore, on days involving intensive field exercises, the energy content of the daily ration should be increased by an additional 500 kcal to meet the elevated energy demands.

## Figures and Tables

**Table 1:** Characteristics of the soldiers participating in the study.

	Special Unit “GROM”	Special Unit of the Military Police (MP)	Average
Age [years]	30.1 ± 3.1	32.5 ± 5.9	31.3 ± 4.6
Body weight [kg]	86.8 ± 10.0	86.9 ± 8.8	86.8 ± 9.2
Height [cm]	179.8 ± 6.6	181.5 ± 5.8 *	180.6 ± 6.2
BMI	26.8 ± 3.4	26.5 ± 2.9	26.6 ± 3.2

* Difference statistically significant at *p* ≤ 0.05.

**Table 2:** Classification of work severity based on energy expenditure values [26].

Degree of Severity of Work	Daily Energy Expenditure—24 h		Energy Expenditure per Work Shift—8 h	
	[kcal]	[MJ]	[kcal]	[MJ]
Light	2300–2800	9.6–11.7	≤500	≤2.1
Moderate	2800–3300	11.7–13.8	500–1000	2.1–4.2
Moderate hard	3300–3800	13.8–15.9	1000–1500	4.2–6.3
Hard	3800–4300	15.9–18.0	1500–2000	6.3–8.4
Very hard	4300–4800	18.0–20.1	2000–2800	8.4–11.7
Extremely hard	≥4800	≥20.1	≥2800	≥11.7

Difference statistically significant at *p* ≤ 0.05.

**Table 3:** Classification of work severity based on energy expenditure in kcal/min [27].

Degree of Work Severity	The Amount of Energy Load
Light	>2.5 kcal/min
Moderate	>5.0 kcal/min
Hard	>7.5 kcal/min
Very hard	>10.0 kcal/min
Extremely hard	>12.5 kcal/min

**Table 4:** Energy expenditure of soldiers during the execution of training tasks over a typical 8-h garrison training day.

	Energy Expenditure [kcal/min]	Heart Rate (Min.)	Heart Rate (Max.)	Heart Rate (Average)	Total Energy Expenditure Over 8 h [kcal]
GROM Unit	3.2 ± 0.8	51.1 ± 8.1	163.0 ± 50.0	79.7 ± 9.2	1521.6
MP Unit	4.3 ± 1.2	60.3 ± 9.0	145.0 ± 27.0	89.6 ± 9.3	2064.0 *

* Difference statistically significant at *p* ≤ 0.05.

**Table 5:** Energy expenditure of soldiers during a typical 8-h field training day (tactical and shooting exercises).

	Energy Expenditure [kcal/min]	Heart Rate (Min.)	Heart Rate (Max.)	Heart Rate (Average)	Total Energy Expenditure Over 8 h [kcal]
GROM Unit	3.9 ± 0.8	55.2 ± 12.0	170.4 ± 33.7	89.0 ± 8.3	1872.0
MP Unit	4.8 ± 1.2	60.0 ± 9.3	115.6 ± 16.6	92.0 ± 9.3	2304.0 *

* Difference statistically significant at *p* ≤ 0.05.

**Table 6:** Daily energy expenditure values during field training of soldiers from the “GROM” unit.

Soldier	Daily Energy Expenditure [kcal]	Heart Rate (Min.)	Heart Rate (Max.)	Heart Rate (Average)
1.	3962.9 ± 712.4	40	217	75 ± 6.4
2.	4517.3 ± 749.2	47	207	82 ± 6.8
3.	4541.8 ± 732.1	45	189	79 ± 7.8
4.	3553.9 ± 732.2	39	144	75 ± 6.4
5.	3615.8 ± 722.1	38	173	73 ± 7.2
6.	3794.4 ± 718.6	44	180	75 ± 6.8
7.	4050.7 ± 736.9	45	141	78 ± 7.2
8.	3687.8 ± 722.4	30	212	76 ± 7.4
9.	5611.7 ± 715.6	25	167	85 ± 7.6
10.	3513.6 ± 744.2	31	157	70 ± 6.2
11.	4216.3 ± 698.8	38	168	79 ± 6.0
12.	4222.1 ± 689.2	48	150	80 ± 7.2
13.	4708.8 ± 722.2	40	211	75 ± 5.8
14.	3816.0 ± 741.0	64	220	98 ± 8.2
15.	3456.0 ± 719.6	43	187	76 ± 6.6
16.	5716.8 ± 723.8	44	180	79 ± 6.2
17.	3024.0 ± 729.2	37	141	92 ± 6.8
18.	5688.0 ± 742.6	48	169	89 ± 5.8
19.	3931.2 ± 729.8	41	160	75 ± 6.6
20.	3830.4 ± 699.2	44	200	75 ± 6.6
21.	4233.6 ± 720.4	39	181	75 ± 6.4
22.	4161.6 ± 719.9	42	214	76 ± 7.6
Average	4175.0 ± 723.7	41.3 ± 7.86	180.4 ± 26.4	79 ± 6.8

**Table 7:** Daily energy expenditure during a typical field training day for soldiers of the Special Unit of the Military Police.

Time of Activity	Duration [min]	Type of Activity	Energy Expenditure [kcal/kg Body Weight]
5:00 a.m.	-	Wake-up	-
5:00 a.m.–6:15 a.m.	75	Morning exercise, hygiene, area tidying	3.46
6:15 a.m.–6:35 a.m.	20	Breakfast	0.82
6:35 a.m.–6:50 a.m.	15	Collecting weapons and equipment	5.06
6:50 a.m.–7:00 a.m.	10	Morning roll call	0.58
7:00 a.m.–10:35 a.m.	215	Patrol	7.97
10:35 a.m.–11:00 a.m.	25	Second breakfast	1.03
11:00 a.m.–2:00 p.m.	180	Urban tactical training	10.04
2:00 p.m.–4:30 p.m.	150	Lunch and post-meal rest	5.30
4:30 p.m.–7:00 p.m.	150	Securing operations and weapon cleaning	8.04
7:00 p.m.–8:30 p.m.	90	Dinner	3.70
8:30 p.m.–8:40 p.m.	10	Evening roll call	0.57
8:40 p.m.–9:00 p.m.	20	Hygiene and free time	1.19
9:00 p.m.–5:00 a.m.	480	Sleep	7.96
Total	1440	-	55.72
Daily energy expenditure of a soldier with an average body weight of 90 kg			5014.8 kcal ± 666.3 kcal

**Table 8:** Proposed basic nutritional standard for soldiers of special units of the Armed Forces of the Republic of Poland.

Energy/Nutrient per Day	Standard
Energy value	4400 kcal *
Protein content	13–15% of total energy
Fat content	<30% of total energy
including:	
Saturated fatty acids	As low as possible, within a diet providing adequate nutrition
Linoleic acid (C18:2 *n*-6, LA)	4% of total energy
Alpha-linolenic acid (C18:3, *n*-3, ALA)	0.5% of total energy
Eicosapentaenoic acid (C20:5 *n*-3, EPA) + docosahexaenoic acid (C22:6 *n*-3, DHA)	250 mg EPA + DHA/day
Carbohydrate content	55–75% of total energy, not less than 550 g
Dietary fiber content	25 g
Vitamin content	†
Vitamin A	900 µg
Vitamin D	15 µg
Vitamin E	
men	10 mg
women	8 mg
Vitamin K	
men	65 µg
women	55 µg
Vitamin C	90 mg
Vitamin B_1_	
men	1.3 mg
women	1.1 mg
Vitamin B_2_	
men	1.3 mg
women	1.1 mg
Niacin	
men	16 mg
women	14 mg
Vitamin B_6_	1.3 mg
Folates	400 µg
Vitamin B_12_	2.4 µg
Biotin	40 µg
Pantothenic acid	5 mg
Choline	
men	550 mg
women	425 mg
Mineral content	†
Calcium	1000 mg
Phosphorus	700 mg
Magnesium	
men	420 mg
women	320 mg
Iron	
men	10 mg
women	18 mg
Zinc	
men	11 mg
women	8 mg
Iodine	150 µg
Selenium	55 µg
Copper	0.9 mg
Fluoride	
men	4 mg
women	3 mg
Manganese	
men	2.3 mg
women	1.8 mg
Molybdenum	65 µg
Potassium	3500 mg
Sodium	1500 mg
Chloride	2300 mg

* On days of intensive field training, it is permissible to increase the energy value of the food ration by 500 kcal, in the form of carbohydrate-based products; † Vitamin and mineral intake values are consistent with the nutritional standards adopted for the Polish population [51].

## Data Availability

The data presented in this study are available on request from the corresponding author. The data is not publicly available due to privacy and ethical restrictions.

## Abbreviations

The following abbreviations are used in this manuscript:

ALA	Alpha-linolenic acid
ANOVA	Analysis of Variance
BIA	Bioelectrical Impedance Analysis
BMI	Body Mass Index
DHA	Docosahexaenoic acid
EPA	Eicosapentaenoic acid
GROM	Special Military Unit “GROM”
HR	Heart rate
ISO	International Organization for Standardization
LA	Linoleic acid
MP	Military Police
NATO	North Atlantic Treaty Organization
VO_2_	Oxygen uptake
